# Excess mortality and associated sociodemographic factors during the COVID-19 pandemic in rural Pune Maharashtra, India: a retrospective data analysis from the Vadu health and demographic surveillance system

**DOI:** 10.1186/s12963-026-00509-x

**Published:** 2026-08-30

**Authors:** Uddhavi Kand, Chodziwadziwa Kabudula, Bharat Choudhari, Tapas Kumar Mohanty, Sandeep Bhujbal, Arunkumar Gondhali, Jean J. H. Bashingwa, Beth A. Tippett Barr, Stephen Tollman, Sanjay Juvekar, Anand Kawade, Dhiraj Agarwal

**Affiliations:** 1 Vadu Rural Health Program, KEM Hospital Research Centre, Pune, India; 2 University of the Witwatersrand, Johannesburg, South Africa; 3 Nyanja Health Research Institute, Salima, Malawi

**Keywords:** COVID-19 mortality, HDSS, India, Socio-demographic factors

## Abstract

**Background:**

Between January 2020 and August 2023, India documented over 450 million confirmed cases of COVID-19 and nearly 0.5 million COVID-19 deaths. While reported mortality rates in India appeared lower than expected, the actual mortality may be higher due to underreporting and disparities in healthcare access. This study aimed to compare mortality before and during the COVID-19 pandemic and identify associated socio-demographic factors in rural Pune, Maharashtra, India using longitudinal population-based data from the Vadu Health and Demographic Surveillance System.

**Methods:**

Individual and mortality data from the Vadu HDSS, India were collected from 2018 to 2021. Descriptive analyses were conducted to examine the variation in mortality by sociodemographic factors during pre-COVID-19 [2018–2019] and COVID-19 [2020–2021] periods. Cox regression with hazard ratios [HR] and 95% confidence intervals [CI] were used to measure the association between socio-demographic factors and mortality.

**Results:**

There were 2039 deaths that occurred between 2018 and 2021, of which 40.6% [828/2039] were female. The overall risk of mortality increased during COVID-19 [HR1.2,95%CI:1.1–1.3], but this varied by sociodemographic characteristics. The risk of death was greater [HR1.8,95%CI:1.5–2.2] among individuals living in single-person households compared to those living in 2–5 member households, and there was a strong trend of increased mortality risk with older age. Widowed individuals had a greater risk of mortality than those who were married [HR2.1,95%CI:1.9–2.4]. Education was strongly protective against mortality; those who had any level of education had a lower risk of dying than those with no education [HR0.4,95%CI:0.3–0.5].

**Conclusion:**

Increased COVID-19 mortality in India was associated with older age, widowhood, and living alone. These findings highlight the need for targeted vaccination, early clinical care, and community outreach for vulnerable populations during future pandemics.

**Supplementary Information:**

The online version contains supplementary material available at https://doi.org/10.1186/s12963-026-00509-x.

## Background

The Coronavirus Disease-2019 [COVID-19] pandemic caused by SARS-COV-2 caused global social and economic disruption, and in many countries presented the greatest challenge yet for public health surveillance and response [1, 2]. Since early 2020, COVID-19 has caused over 6.9 million deaths, with over 7.71 billion confirmed cases till August 2023 globally [3]. India, the world’s most populous country, faced a severe burden of disease, reporting over 450 million confirmed cases and nearly 0.5 million deaths between January 2020 and August 2023 [4]. It is assumed that, actual mortality in India may have been higher due to underreporting and disparities in healthcare access, particularly in rural and underserved areas [5, 6]. Existing socio-economic disparities and high population density contributed to the rapid spread of COVID-19, with the second highest number of cases documented globally [4]. As of July 2023, India reported 0.5 million deaths, with over 450 million confirmed cases [as per World Health Organisation weekly-epidemiological-update-on-covid-19–27-July-2023].

Mortality is a multifactorial event influenced by socio-demographic characteristics, individual health and clinical comorbidities, and environmental conditions [7–9]. In a diverse country like India, where health priorities vary from state to state, a better understanding of these socio-demographic factors can help prioritize patient needs and care; and improve preventive care, including vaccination initiatives [10].

The Vadu Health and Demographic Surveillance System [HDSS] provides a unique platform to examine mortality patterns in a well-defined rural population over time. The HDSS monitors all individuals residing within its surveillance area through regular household visits, conducted biannually or annually, to capture vital events including births, deaths, migrations, and changes in marital status [11]. This longitudinal, population-based surveillance generates high-quality socio-demographic and mortality data, enabling the assessment of mortality trends and their associated determinants across different time periods [12]. Because these data were collected continuously before and throughout the COVID-19 pandemic, the Vadu HDSS offers a reliable baseline for comparing mortality patterns during the pre-pandemic and pandemic periods [13].

Longitudinal mortality data from rural India remain limited. The Vadu HDSS provides continuous population-based surveillance of vital events in a well-defined rural population, making it well suited to assess mortality patterns before and during the COVID-19 pandemic. Using mortality data collected between 2018 and 2021, this study aimed to compare mortality before and during the COVID-19 pandemic and identify socio-demographic factors associated with mortality using longitudinal data from the HDSS in rural Pune, Maharashtra, India [14].

## Methods

### Study design

We conducted a retrospective cohort study using data from the Vadu HDSS in rural Pune, Maharashtra, India. The Vadu HDSS is a well-established longitudinal surveillance system that covers a defined population in a specific geographic region. This study mortality trends over a four-year period from 2018 to 2021, divided into pre-COVID-19 [2018–2019] and COVID-19 pandemic [2020–2021] periods.

### Study setting [area and population]

**Fig. 1:**
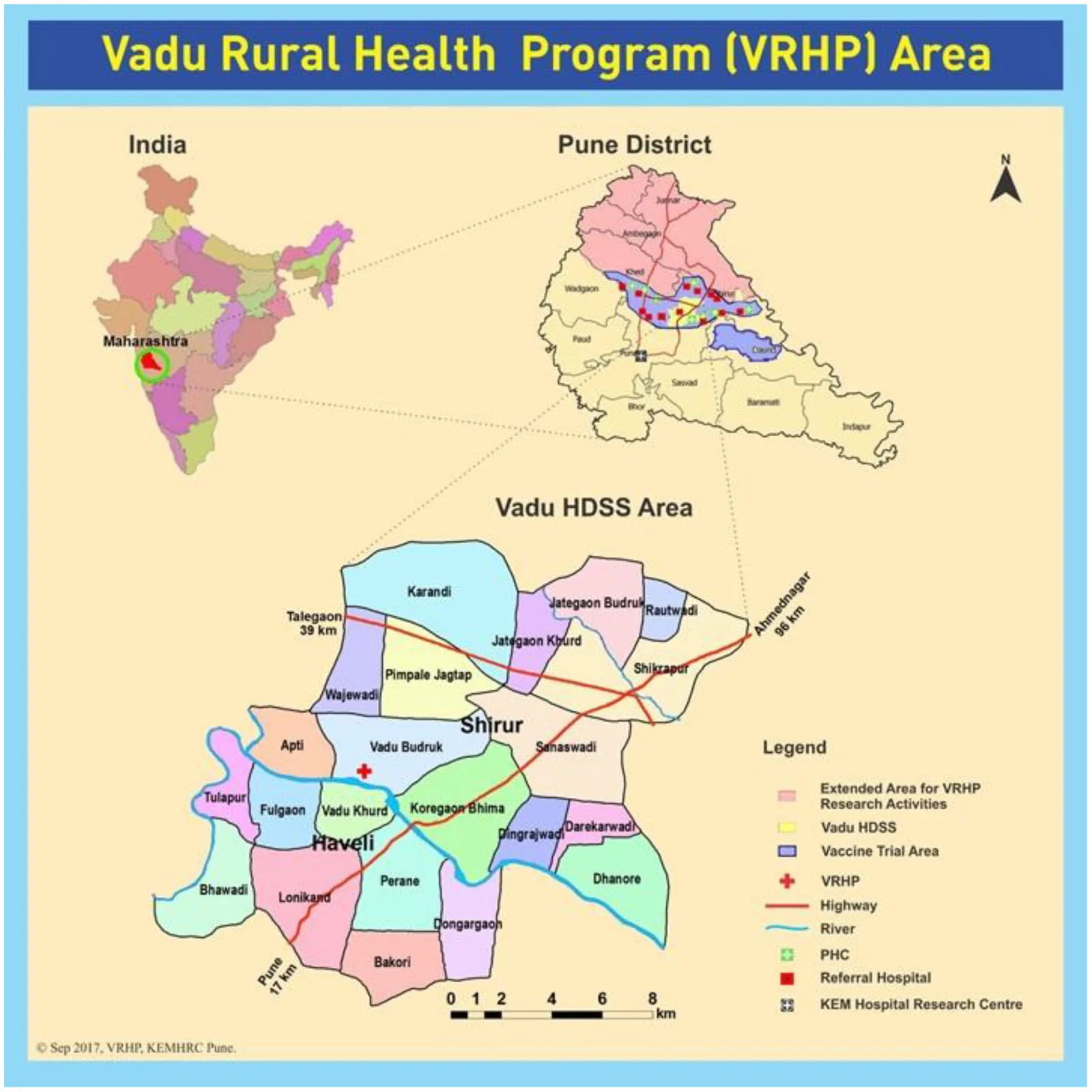
Vadu rural health Program [VRHP] area

The study setting is shown in Fig. 1, which provides a clear geographic representation of the study area. The Vadu HDSS was established by the Vadu Rural Health Program, KEM Hospital Research Centre, Pune in year 2002, to conduct longitudinal population surveillance in rural Pune district. The surveillance area comprises of 22 villages within two blocks Shirur and Haveli of Pune district that were purposively selected during establishment based on feasibility of long-term surveillance. The HDSS follows all resident households through annual surveillance rounds recording births, deaths, pregnancies and migrations [14]. The area is defined as rural but some villages near the highway are taking the shape of peri-urban settings due to industrialisation [14]. As of 2023, the Vadu HDSS covered approximately 2,00,000 individuals residing in 62,000 households. The demographic breakdown was as follows: 27% of the population aged 0–14 years, 68% between 15–59 years, and 5% were aged 60 years and above. 56% of the population was male, and 44% was female. The majority of residents were engaged in agriculture, daily-wage labor, and small-scale business activities. A smaller proportion worked in industrial sector due to the proximity of nearby urban centers. While primary and secondary education was widely available, access to higher secondary and tertiary education was limited within the study area, requiring students to travel to nearby towns and cities. The area is served by one Rural Hospital [secondary care], two Primary Health Centers [PHCs], several sub-centers, and multiple private clinics. Despite the presence of these facilities, access to specialized care remained a challenge, particularly for chronic diseases such as hypertension, diabetes, and respiratory illnesses. The region reports seasonal outbreaks of infectious diseases such as dengue and malaria. Additionally, non-communicable diseases [NCDs] contribute significantly to the local disease burden.

During the COVID-19 pandemic, the region experienced two major waves:

1. First wave [April 2020 – January 2021]
2. Second wave [March 2021 – June 2021]

The pandemic led to substantial changes in healthcare utilization, mortality trends, and socioeconomic conditions, making the Vadu HDSS an ideal setting for assessing the impact of COVID-19 on mortality [15].

### Study participants

The study population included all individuals who were part of the Vadu HDSS during the study period, from 2018 to 2021.

### Data collection

#### Training and quality check

Data collection at Vadu HDSS is performed by trained field research assistants [FRAs], who are local residents of the Vadu HDSS area. These FRAs undergo comprehensive training on standardised data collection methods, ethical considerations and technological literacy. To maintain data quality, a two-step data quality check is implemented. First, the quality control team reviews the collected data for completeness and consistency. Any discrepancies or issues are addressed promptly in collaboration with the FRAs. After the data is collected, it is uploaded onto the servers. Data managers along with research scientists perform a second round of quality checks applying automated scripts to validate the accuracy and reliability of the uploaded data. Before analysis, duplicate records, missing data points, and inconsistencies were addressed through cross-verification with previous data rounds.

#### Pre-pandemic data collection and sources

We collected data from all residents and those who migrated to the area with an intention to stay for more than three months. Data were collected using the Survey Solution data collection application [The World Bank, 2018 *Survey Solutions CAPI/CAWI platform: Release 5.26*. Washington DC, The World Bank] which was customised per our requirements. All households in Vadu HDSS area were visited once a year during which data were collected on births, deaths, marriage, migrations, pregnancy status and outcomes of every woman in reproductive age group, and self-reported common illnesses. Verbal Autopsy [VA] was conducted for every death, a method used to determine the probable cause of death when a person dies outside a health facility and no medically certified cause of death is available. It is a structured interview conducted with the closest caregiver or family member of the deceased to obtain information regarding symptoms, circumstances and events preceding death to understand causes of death.

#### Data collection during COVID-19 period

Due to the pandemic, door-to-door data collection was stopped. Due to movement restrictions and local lock down norms, we collected data telephonically from each household in the catchment area during the pandemic period [2020 and 2021].

### Data management

**Fig. 2:**
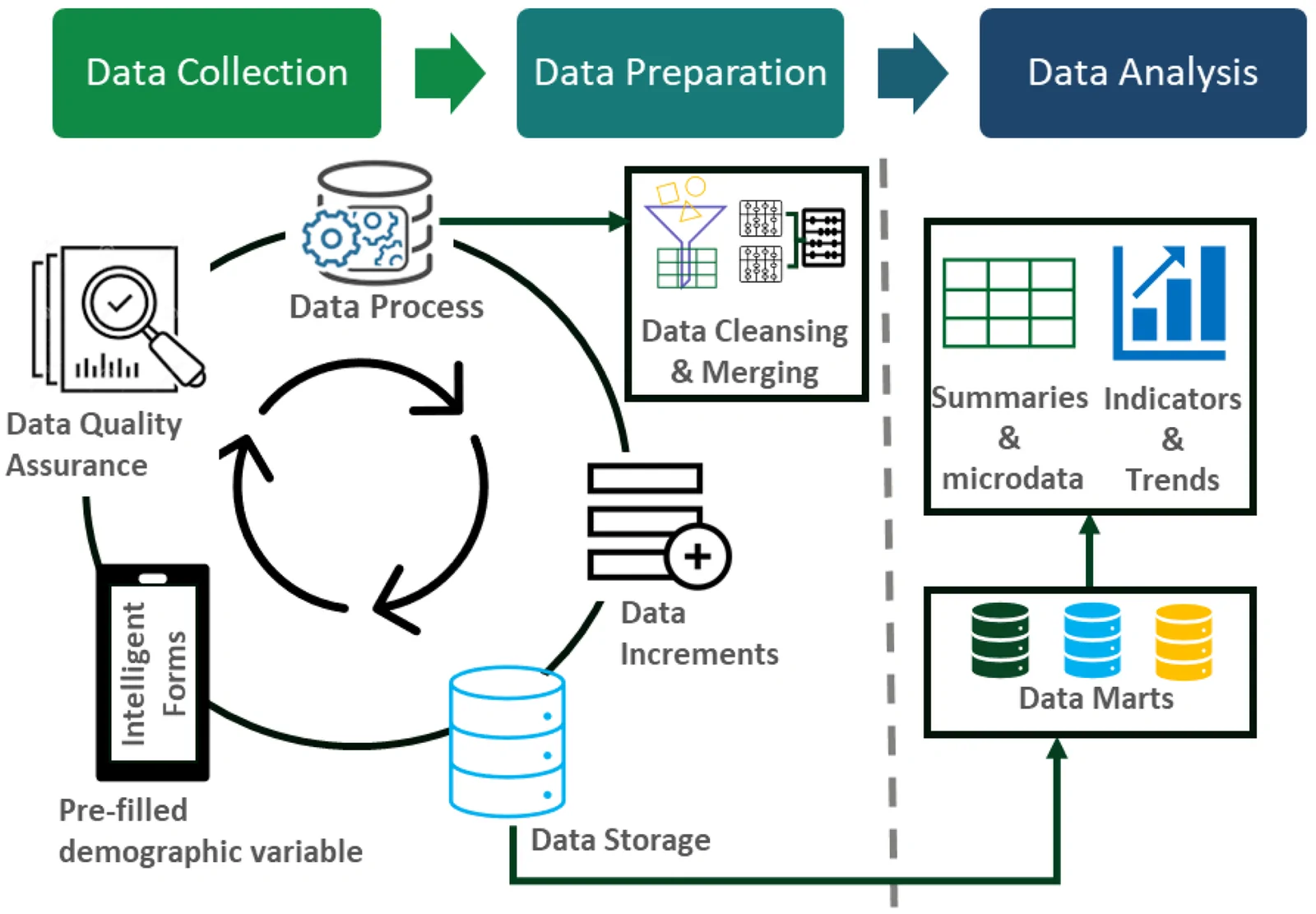
Data collection, management, and analysis of Vadu HDSS

HDSS data is collected by the FRAs on android-based tablet/mobile offline and is synchronised with the office data server once the FRA connects to the internet. Data is retrieved once data synchronisation is complete. Original file of retrieved data is stored by the data manager and IT-team. Data retrieved is processed for any discrepancies, inconsistencies, and missing information, constituting the first step of quality check. After the first step, the quality assurance team completes detailed quality check. Discrepancies in data are marked and file is sent back to the FRA for cross-checking and verification. Figure 2 describes the data flow of Vadu HDSS.

### Data cleaning and analysis

#### Datasets

Before conducting the analysis, all datasets were collated and cleaned to ensure there were no duplicates and data entry errors, and to ensure consistency in data formats using Stata version 15 [StataCorp, 2020, College Station, TX]. Individual data and mortality data were merged for the analysis.

#### Data variables

Deaths that occurred between 2018 to 2021 were included as an outcome variable. Socio-demographic characteristics included age groups [0–14, 15–29, 30–44, 45–59, and 60+ years of age], sex [male or female], education level [pre-primary, completed primary, completed secondary, completed higher secondary, completed graduation, completed post-graduation, and illiterate], marital status [married, remarried, separated, divorced, widow/widower, and unmarried], and household size. Data were collected using the standardized Vadu Health and Demographic Surveillance System (HDSS) death data collection form. The form used for routine death surveillance is provided as Additional file 1.

#### Descriptive analysis

Descriptive analyses were performed to quantify deaths occurring in each year, described by sociodemographic characteristics, from which crude death rates were calculated. We tested the proportional hazards assumption to ensure the validity of the model. This was assessed using a non-significant result [*p* > 0.05] and confirmed that the proportional hazards assumption was met for all included variables. Variables for the final adjusted Cox model were selected based on their significance in univariate analysis [*p* < 0.20]. Model fitness was evaluated using the Akaike Information Criterion [AIC] to identify the best-fitting model. Additionally, concordance [C-index] was calculated to assess the predictive accuracy of the model. A C-index value closer to 1.0 indicated a good predictive ability. Analysis was performed using the Stata [Release 15, 2017].

#### Cox regression with hazard ratios

Cox regression with hazard ratio [HR] and 95% confidence intervals [CI] was used to assess the association between socio-demographic factors and mortality. HR were calculated for each socio-demographic variable including age, gender, marital status, education. To explore the association between each socio-demographic factors and the mortality, we performed univariate and multivariate analysis cox proportional hazards model. Results were reported as HRs, along with their 95% CIs and corresponding *p*-values. Socio-demographic factors with a *p*-value < 0.05 in the univariate analysis were considered for inclusion in the multivariate analysis. The descriptive analyses presented in this study were based on the mortality dataset and were used to summarize the characteristics of recorded deaths and calculate crude death rates. For the Cox proportional hazards analysis, mortality records were linked with the longitudinal HDSS database to estimate mortality risk according to socio-demographic characteristics.

### Age and gender specific mortality rates

Age and gender specific mortality rates were calculated from 2018 to 2021. The crude death rate [CDR] was calculated as the number of deaths during the specified period divided by the total person-years under surveillance, multiplied by 1,000, and expressed as deaths per 1,000 person-years.

## Results

### Demographic characteristics of the population

Table 1 shows that from 2018 to 2021, there were 2039 deaths. Of these, 1211 [59.4%] were males and 828 [40.6%] were females. The pre-COVID-19 crude death rate was 3.8 [95%CI:3.5–4.1] per 1000 persons [975 deaths, 260,379 person-years], which increased to 4.4 [95%CI:4.1–4.8] per 1000 persons during the pandemic [1064 deaths, 241,807 person-years].

**Table 1:** Crude death rate [CDR] per 1,000 person-years by sociodemographic characteristics during pre-COVID-19 and COVID-19 period, Vadu, India, 2018–2021

	Pre-COVID-19 Period [2018–2019]				COVID-19 Period [2020–2021]			
	Alive	Deaths	Person-years	CDR per 1000 [95%CI]	Alive	Deaths	Person-years	CDR per 1000 person-years
Age group								
0–14	38,870	49	75,315	0.7 [0.5–0.9]	35,823	20	70,863	**0.3 [0.2–0.4]**
15–29	47,903	48	80,200	0.6 [0.4–0.8]	40,400	39	62,919	0.6 [0.4–0.8]
30–44	33,591	98	62,113	1.6 [1.3–1.9]	38,283	128	63,005	2.0 [1.7–2.4]
45–59	13,895	179	27,135	6.6 [5.7–7.6]	15,826	215	27,935	7.7 [6.7–8.8]
60+	7,749	601	15,976	37.6 [34.7–40.8]	9,507	662	17,085	38.8 [35.8–41.7]
Gender								
Male	79,704	574	1,40,107	4.1 [3.7–4.4]	78,109	637	1,25,996	**5.1 [4.6–5.4]**
Female	62,304	401	1,20,632	3.3 [3.0–3.6]	61,730	427	1,15,811	3.7 [3.3–4.0]
Education								
No formal education	21,678	556	76,114	7.3 [6.7–7.9]	17,504	561	70,334	8.0 [7.3–8.7]
Primary	55,380	198	62,207	3.2 [2.8–3.7]	52,767	261	57,300	**4.6 [4.0–5.1]**
Secondary	43,541	123	74,981	1.6 [1.4–2.0]	44,148	166	69,171	**2.4 [2.1–2.8]**
Graduate and higher	13,938	20	24,374	0.8 [0.5–1.3]	14,405	36	22,362	1.6 [1.2–2.2]
Unknown	7,471	78	23,063	3.4 [2.7–4.2]	11,015	40	22,640	**1.8 [1.3–2.4]**
Marital Status								
Unmarried	54,010	52	86,389	0.6 [0.5–0.8]	49,384	36	75,295	0.5 [0.3–0.7]
Married	71,274	578	1,32,183	4.4 [4.0–4.7]	69,727	704	1,26,207	**5.6 [5.2–6.0]**
Divorced/separated	407	1	726	1.4 [0.2–9.8]	396	8	657	12.2 [6.1–24.4]
Widowed	4,465	262	8,122	32.3 [28.6–36.4]	4,277	274	7,290	37.6 [33.4–42.3]
Unknown	11852	82	33,319	2.5 [2.0–3.1]	16055	42	32,357	**1.3 [1.0–1.80]**
Household size								
1	17,843	78	18,668	4.2 [3.3–5.2]	17,723	69	9,597	**7.2 [5.7–9.1]**
2–5	26,788	305	1,28,984	2.3 [2.1–2.70]	25,382	319	1,20,896	2.6 [2.4–2.9]
>5	4,293	592	1,13,087	5.2 [4.8–5.7]	4,334	676	1,11,314	6.1 [5.6–6.5]

Figures in bold experienced change during COVID-19 at p < 0.05 significance

### Crude death rates and socio-demographic characteristics

During the pre-COVID-19 period [2018–2019], the crude death rate [CDR] ranged from 0.7 [95%CI:0.5–0.9] per 1,000 persons in the 0–14 age group to 37.6 [95%CI:34.7–40.8] per 1,000 persons in the 60+ age group. The CDR during the COVID-19 period [2020–2021], showed an increase in all age groups compared with pre-COVID-19 period, except the 0–14 years age group which showed a decrease to 0.3 [95%CI:0.2–0.4] per 1,000 person years. The Highest CDR during the COVID-19 period of 38.8 [95%CI35.8–41.7] per 1,000 person years was observed compared with 37.6 [95% CI: 34.7–40.8] per 1,000 person-years during the pre-COVID-19 period in the 60+ age group Table 1.

The CDR for males was 4.1 [95%CI:3.7–4.4] per 1,000 persons in the pre-COVID-19 period which increased to 5.1 [95%CI:4.6–5.4] per 1,000 person years during the COVID-19 period. Among females, the CDR was 3.3 [95%CI:3.0–3.6] per 1,000 person years during the pre-COVID-19 period and rose to 3.7 [95%CI:3.3–4.0] per 1,000 person years during the COVID-19 period. Individuals with lower levels of education generally displayed higher CDR.

Individuals with no formal education had an increase in CDR in the pre-COVID-19 period from 7.3 [95%CI:6.7–7.9] per 1000 person years to 8.0 [95%CI:7.3–8.7] per 1000 person years during the COVID-19 period. Similarly, the primary education group’s CDR rose from 3.2 [95%CI:2.8–3.7] to 4.6 [95%CI:4.0–5.1] per 1000 person years. Widowed individuals experienced an increase in CDR in the pre-COVID-19 period from 32.3 [95%CI:28.6–36.4] to 37.6 [95%CI:33.4–42.3] per 1,000 person years during the COVID 19 period. CDR of married individuals increased in the pre-COVID-19 period from 4.4 [95%CI:4.0–4.7] per 1,000 person years to 5.6 [95%CI:5.2–6.0] per 1,000 person years during the COVID-19 period [Table 1].

Households with only one member witnessed a notable increase in CDR in the pre-COVID-19 period from 4.2 [95%CI:3.3–5.2] to 7.2 [95%CI:5.7–9.1] per 1,000 person years during the COVID-19 period. Households with more than 5 members had a CDR in the pre-COVID-19 period of 5.2 [95%CI:4.8–5.7] per 1,000 person years, which increased to 6.1 [95%CI:5.6–6.5] per 1,000 person years during the COVID-19 period [Table 1].

### Mortality hazard

**Fig. 3:**
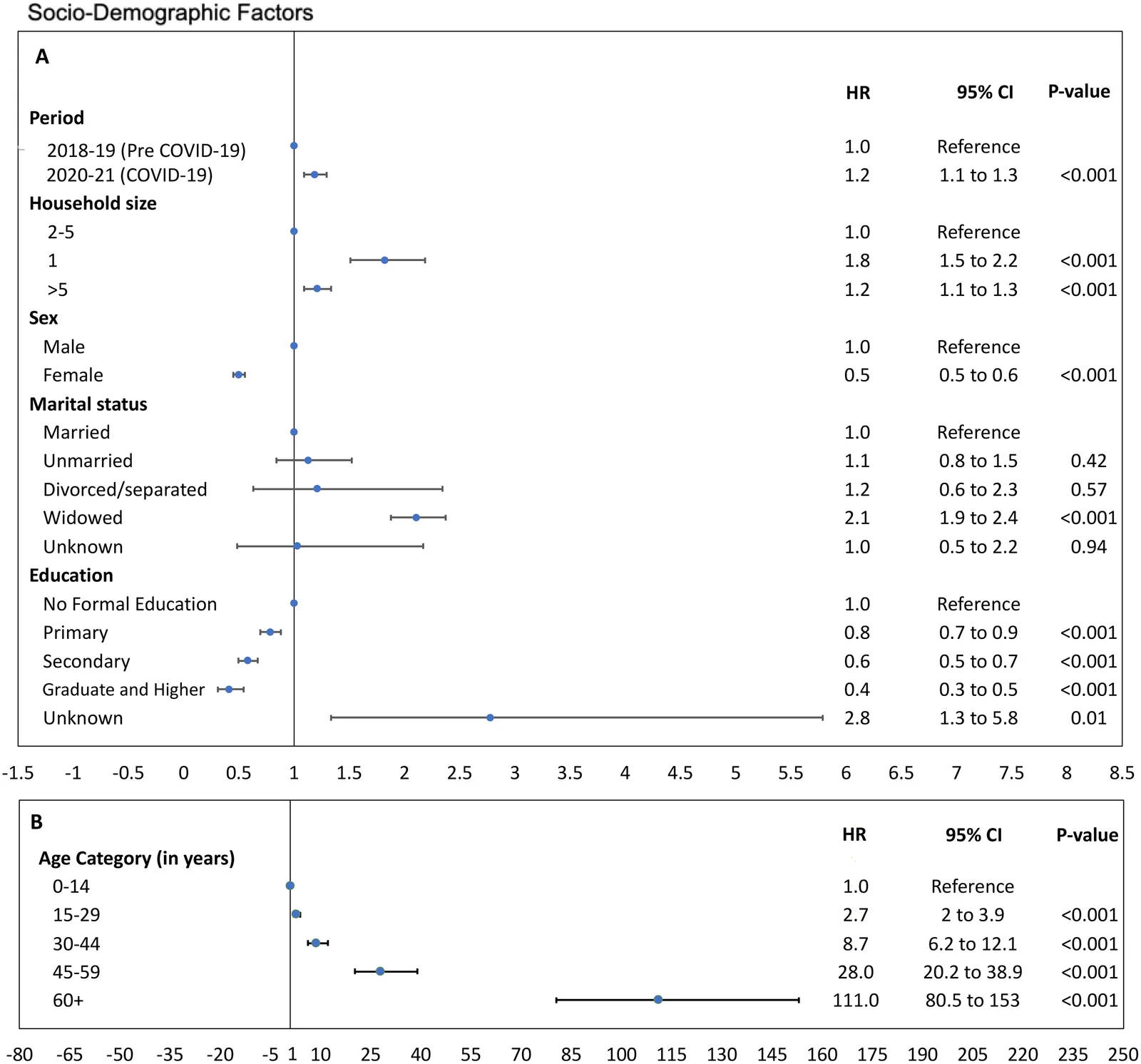
Socio-demographic factors associated with mortality, results from cox proportion hazards regression model, Vadu HDSS, India, 2018–2021

Cox regression multivariate analysis was conducted to identify socio-demographic factors associated with mortality in the Vadu HDSS population during 2018–2021 [Fig. 3], revealing a strong association between age and mortality [*p* < 0.001]. Compared to individuals aged 0–14 years [reference group], the hazard of death was higher in all other age groups [15–29 age group:HR2.75,95%CI:1.96–3.86, 30–44 age group: HR8.67,95%CI:6.19–12.15, 45–59 age group HR28.02,95%CI:20.18–38.92, and the 60+ age group: HR111.01,95%CI:80.55–152.98]. The HR for females was [HR0.49,95%CI:0.45–0.55], compared with males [reference group], indicating a lower risk of mortality compared to males [*p* < 0.001]. In comparison with individuals with no formal education individuals [reference group] with higher education levels had lower hazard of death. The HR were 0.78[95%CI:0.69–0.88] for primary education, 0.5 [95%CI:70.49–0.67] for secondary education, and 0.40 [95%CI:0.30–0.54] for graduate and higher education [*p* < 0.001]. In comparison with married individuals [reference group], widowed individuals were at a higher risk of mortality [HR2.11,95%CI:1.87–2.37,*p* < 0.001]. Individuals in single-person households had an increased hazard of death [HR1.81,95%CI:1.51–2.18,*p* < 0.001], while those in larger households with more than 5 members also exhibited a slightly elevated risk [HR1.20,95%CI:1.09–1.33,*p* < 0.001], compared to households with 2–5 members [reference group]. Findings from univariate Cox analysis were consistent with those from multivariate analysis with respect to association of socio demographic factors at associated with mortality during the pre-COVID-19 [2018–2019] and COVID-19 [2020–2021] periods [Table 2].

**Table 2:** Univariate and multivariate analysis of socio-demographic factors associated with excess mortality during COVID-19 pandemic

	Univariate Analysis				Multivariate Analysis			
Socio-Demographic Factors	HR	Lower CI	Upper CI	p-value	HR	Lower CI	Upper CI	p-value
Period								
2018–19 [Pre COVID-19]	1.0				1.0			
2020–21 [COVID-19]	1.2	1.1	1.4	0.00	1.2	1.1	1.3	<0.001
Household size								
2–5	1.0				1.0			
1	2.1	1.7	2.5	0.00	1.8	1.5	2.2	<0.001
>5	2.3	2.0	2.5	0.00	1.2	1.1	1.3	<0.001
Sex								
Male	1.0				1.0			
Female	0.8	0.7	0.8	0.00	0.5	0.5	0.6	<0.001
Marital status								
Married	1.0				1.0			
Unmarried	0.1	0.1	0.1	0.00	1.1	0.8	1.5	0.42
Divorced/separated	1.3	0.7	2.5	0.42	1.2	0.6	2.3	0.57
Widowed	7.0	6.3	7.7	0.00	2.1	1.9	2.4	<0.001
Unknown	0.4	0.3	0.5	0.00	1.0	0.5	2.2	0.94
Education								
No Formal Education	1.0				1.0			
Primary	0.5	0.5	0.6	0.00	0.8	0.7	0.9	<0.001
Secondary	0.3	0.2	0.3	0.00	0.6	0.5	0.7	<0.001
Graduate and Higher	0.2	0.1	0.2	0.00	0.4	0.3	0.5	<0.001
Unknown	0.3	0.3	0.4	0.00	2.8	1.3	5.8	0.01
Age Category [in years]								
0–14	1.0				1.0			
15–29	1.3	1.0	1.8	0.099	2.7	2.0	3.9	<0.001
30–44	3.8	2.9	5.0	0.00	8.7	6.2	12.1	<0.001
45–59	15.1	11.7	19.6	0.00	28.0	20.2	38.9	<0.001
60+	80.8	63.4	103.0	0.00	111.0	80.5	153.0	<0.001

## Discussion

This study provides comprehensive analysis of mortality trends of different socio-demographic characteristics such as age, gender, education level, marital status, and household size. during the COVID-19 pandemic in the Vadu HDSS region of India. These results are in line with national and global research of COVID-19 impact on vulnerable population [15–18]. Mortality risk increased significantly with age [HR 2.75 for 15–29 years, HR 8.67 for 30–44 years, HR 28.02 for 45–59 years, and HR 111.01 for 60+ years]. Males were at greater risk of death during the pandemic compared to pre-COVID-19 and compared to females. People with no education were at higher risk of dying during the pandemic compared to all education groups. Additionally, widowed individuals were at greater risk of death during COVID-19 than other groups. Households with single member and families with more than 5 individuals showed significant risk of mortality during COVID 19.

The markedly higher mortality among older adults observed in our study is consistent with previous reports from India and other countries [15–19]. While mortality naturally increases with age, the substantially elevated hazard among adults aged ≥ 60 years during the study period likely reflects the combined effects of age-related frailty, a higher prevalence of chronic conditions, and increased vulnerability to severe COVID-19. These findings underscore the importance of ensuring uninterrupted access to preventive and curative healthcare for older adults during public health emergencies, including timely vaccination, management of chronic diseases, and early access to treatment. Our study observation that males were at higher risk and showed higher mortality rates, finds parallels in research by others. Other studies noted that despite the presence of the COVID-19 pandemic, males had higher mortality [17–19].

Our findings concur with previous research which shows that COVID-19 related mortality is 1.4 to 1.9-fold greater among low education status, thus indicating a connection between lower education levels and higher mortality [20–22]. This association suggests that education plays a vital role in shaping health-related behaviours, access to healthcare, and overall health awareness. The mortality rate in the uneducated was more than twice of other education categories, with confounding in the 60+ age group, where the mortality rate is highest due to age as well. Similar to our finding, other research shows increased mortality among widowed individuals [21, 23, 24]. This consistent pattern suggests that the loss of a spouse can lead to psychosocial stressors that impact overall health and mortality.

Our study’s identification of increased mortality risks in single-member households and larger households resonates with the findings of the study in Chennai, India, which showed identical corelation with household crowding [17]. A study in Los Angeles also showed that there was positive association between an overcrowded house and COVID 19 mortality [25]. Although another study in US showed no associations between households with more than four persons and hospitalization for COVID-19 symptoms, compared to one-person households [26], it points towards the potential challenges in managing health and exposure in large household during the COVID-19 pandemic.

The Sample Registration System [SRS] data for Maharashtra during 2019 –2020 reports the CDR to be about 5.5 per 1000 population [6.2 in rural areas]. Our data analysis showed, during the pre-COVID-19 CDR were 3.8 per 1000 population which increased to 4.4 per 1000 persons during the pandemic. The reason for this could be the HDSS conducts routine annual data collection by visiting every household in the study area. This rigorous approach allows for the systematic capture of all vital events, including deaths. The SRS, which uses sampling to estimate vital rates for a large and diverse population, the HDSS follows the same population longitudinally, ensuring consistency in data collection and reducing biases. Additionally, the HDSS focuses on a smaller, well-defined population that may differ in demographic and socioeconomic characteristics from the state-wide population sampled by SRS, which justifies the differences in the CDRs from both sources. The consistent quality assurance process, pre- and during the pandemic, suggests that the observed increase in CDR reflects genuine trends

The results of this study carry several important implications for public health policy and interventions in the Vadu region. Firstly, targeted efforts to protect vulnerable populations during epidemics, such as older adults, males and people with no education, should be prioritised [27]. Health authorities should consider tailored awareness campaigns and vaccination strategies to mitigate risks for these groups [28]. Secondly, addressing health inequities is crucial to improve overall pandemic response. Socioeconomic support and access to healthcare services should be prioritised for individuals in the high risk groups [29]. Health authorities should allocate resources strategically, taking into account geographical variations and the density of COVID-19 cases in different areas [30]. Moreover, understanding the socio-demographic patterns of COVID-19 deaths can aid in predicting vulnerabilities in future outbreaks and guiding preparedness efforts.

Our study benefits from utilising data from the Vadu HDSS, a well-established surveillance system that provides comprehensive information on the population’s health and demographic characteristics. The long study period from 2018 to 2021 allowed for a comprehensive analysis of trends before and during the pandemic. This manuscript highlights the potential to leverage HDSS tools and methods for data triangulation with the Civil Registration System [CRS] to enhance the completeness and reliability of mortality reporting.

The findings of this study have important implications for public health preparedness and pandemic response in rural India. The observed increase in mortality during the COVID-19 period, particularly among older adults, widowed individuals, people living alone, and those with lower educational attainment, highlights the need for targeted interventions to protect socially and medically vulnerable populations during public health emergencies. Strengthening community-based surveillance systems such as the Vadu HDSS, ensuring uninterrupted access to essential healthcare services, promoting timely vaccination, and implementing targeted risk communication and social support programmes may help reduce excess mortality during future epidemics. Continued longitudinal surveillance is essential for monitoring mortality trends, identifying emerging vulnerable groups, and generating evidence to inform equitable and evidence-based public health policies.

## Limitations

However, this study has several limitations and should be considered when interpreting the findings. Although verbal autopsy interviews were completed for all recorded deaths, cause-of-death assignment was still ongoing at the time of analysis; therefore, this study examined all-cause mortality rather than cause-specific mortality. The Vadu HDSS represents a rural population in Pune district, Maharashtra, and the findings may not be generalisable to other regions of India with different demographic, socioeconomic, and healthcare characteristics. The retrospective cohort study relied on routinely collected surveillance data, which may be subject to incomplete or missing information despite established quality assurance procedures. The analysis focused primarily on socio-demographic characteristics and did not include important clinical and contextual factors such as pre-existing comorbidities, healthcare utilisation, COVID-19 vaccination status, or healthcare access, all of which may have influenced mortality risk. In addition, socioeconomic indicators such as household income, occupation, and household wealth were not available for analysis, leaving the possibility of residual confounding. Another limitation is that the descriptive analyses presented in this manuscript are based primarily on the mortality dataset. Consequently, detailed baseline characteristics of individuals who remained alive during the study period were not presented, although all individuals under surveillance contributed person-time to the Cox proportional hazards model until death, migration, or the end of follow-up.

Future research should integrate verbal autopsy-derived causes of death with socioeconomic and clinical information to better understand the pathways contributing to excess mortality during pandemics. Such evidence will support more targeted preparedness strategies and strengthen resilience to future public health emergencies

## Conclusions

These findings underscore the importance of implementing targeted public health interventions to address the excess mortality associated with the COVID-19 pandemic. Strategies should focus on protecting vulnerable populations, ensuring access to healthcare services, and promoting equitable distribution of resources. Continued monitoring and analysis of excess mortality rates is crucial for informing evidence-based policies and interventions to detect emerging health threats early enough and guide rapid response strategies for future pandemics.

## Electronic supplementary material

Below is the link to the electronic supplementary material.


Supplementary material 1: Vadu Health and Demographic Surveillance System (HDSS) death data collection form used for routine mortality surveillance and data collection


## Data Availability

*Data used in this paper is made available on GitHub.* The data can be accessed using the link: [https://github.com/SAPRIN-MRC/ExcessMortality.input/tree/e3946601af8f3c5335bd57c7ed781d4e93b7bfe3/IN021]

## Abbreviations

CDR: Crude death rate
CI: Confidence Interval
COVID-19: Coronavirus Disease 2019
FRA: Field Research Assistant
HDSS: Health and Demographic Surveillance System
HR: Hazards Ratio
VA: Verbal Autopsy
