# Supplementary material for: Excess mortality and associated sociodemographic factors during the COVID-19 pandemic in rural Pune Maharashtra, India: a retrospective data analysis from the Vadu health and demographic surveillance system
Source: Popul Health Metr. 2026 Aug 30;23(Suppl 2):81. doi: 10.1186/s12963-026-00509-x (PMC13536605; doi:10.1186/s12963-026-00509-x)
Supplement: Supplementary file 1 [file 12963_2026_509_MOESM1_ESM.pdf]

KEM Hospital Research Centre Pune, Vadu Rural Health Program (VRHP)
Vadu Health and Demographic Surveillance System (Vadu HDSS)

Death Event Form

Interview start time मुलाखत सुरु करण्याची वेळ: □□:□□ *(24 hrs)*		
PID<br>□□□ □□□ □□□□ □□		Current Status<br>□□□ □□□ □□□□ □□
Q0001	Household number<br>घर क्रमांक	□□□ □□□ □□□□<br>Village गाव Wadi वाडी Household number घर क्रमांक
Q0002	Does this deceased person permanently belong to Vadu HDSS area? ही मृत व्यक्ती कायमची वढू एचडीएसएस परिसरातील आहे का?	01 Resident of Vadu HDSS area वढू एचडीएसएस परिसरातील रहिवासी<br>02 Visitor to Vadu HDSS area *(to any household)* वढू एचडीएसएस परिसराला भेट देणारे (कोणत्याही घराला*)*<br>03 Person travelling through Vadu HDSS area *(go to Q0005)* वढू एचडीएसएस परिसरातून प्रवास करणारी व्यक्ती *(Q0005 वर जा)*
Q0003	Name of head of the household<br>कुटुंब प्रमुखाचे नाव<br>Village name गावाचे नाव<br>Wadi name वाडीचे नाव<br>Landmark घराजवळील खूण<br>Phone number फोन नंबर	________________<br>________________<br>________________<br>________________<br>________________
Q0004	Name of the respondent *(Respondent should be head of the household)*<br>उत्तर देणाऱ्या व्यक्तीचे नाव:<br>*(उत्तर देणारी व्यक्ती हि कुटुंबातील प्रौढ व्यक्ती असली पाहिजे)*	________________
Q0005	Name of the deceased person<br>मृत व्यक्तीचे नाव	________________
Q0006	Permanent address of the deceased person मृत व्यक्तीचे नेहमीचे राहण्याचे ठिकाण	Village name गावाचे नाव ________________

Death Event Form

		Wadi name वाडीचे नाव ________________
Q0007	Place where death occurred<br>मृत्यूचे ठिकाण	Village name गावाचे नाव ________________<br><br>Wadi name वाडीचे नाव ________________
Q0008 A	Age of the deceased person in years<br>मृत व्यक्तीचे (मृत्यूच्या वेळेचे) वय **(वर्षे)**	☐☐ Years वर्षे
Q0008 B	Age of the deceased person in months<br>मृत व्यक्तीचे (मृत्यूच्या वेळेचे) वय **(महिने)**	☐☐ Months महिने
Q0008 C	Age of the deceased person in days<br>मृत व्यक्तीचे (मृत्यूच्या वेळेचे) वय **(दिवस)**	☐☐ Days दिवस
Q0009	Sex of the deceased person<br>मृत व्यक्तीचे लिंग	01 Male स्त्री<br>02 Female पुरुष<br>03 Other इतर
Q0010	Occupation of the deceased person<br>मृत व्यक्तीचा व्यवसाय	
Q0011	Marital status of the deceased person<br>मृत व्यक्तीची वैवाहिक स्थिती	01 Married विवाहित<br>02 Re-married *(widow/widower or second marriage when husband/wife is alive)* पुनर्विवाहीत *(विधवा/विधुर आहे किंवा नवरा/बायको जिवंत असताना)*<br>03 Separate विभक्त<br>04 Divorced घटस्फोटित<br>05 Widow/widower विधवा/विधुर<br>06 Unmarried अविवाहित
Q0012	What was the relationship of the deceased person with you?<br>मृत व्यक्तीचे तुमच्या सोबत नाते काय होते?	~~01 Self स्वतः~~<br>02 Spouse *(wife/husband)* जोडीदार (पत्नी/पती)<br>03 Son/Daughter (मुलगा/ मुलगी)<br>04 Daughter-in -law/son-in-law (सुन/ जावई)<br>05 Grandson/Grand-daughter (नातू/ नात)<br>06 Mother/father (आई/ वडील)<br>07 Mother-in-law/Father-in-law (सासू/ सासरे)<br>08 Brother/Sister (भाऊ/बहीण)<br>09 Step/Co wife (सवत)

Death Event Form

		10 Grandmother/Grandfather (आजी/ आजोबा)<br>11 Other *(Specify)* इतर (नमूद करा)<br>12 No relation but friends/servants/paying guest कोणतेही नाते नाही (मित्र/ नोकर/ पेइंग गेस्ट)<br>~~88 Do not know/cannot say माहित नाही/ सांगू शकत नाही~~
Q0013	Place of death<br>मृत्यू झाला ते ठिकाण	01 Government hospital सरकारी हॉस्पिटल<br>02 Private hospital खाजगी हॉस्पिटल<br>03 Home घरी<br>04 In transit towards hospital हॉस्पिटलकडे जाताना<br>05 Other *(specify)* इतर *(स्पष्ट करा)*
Q0014	Date of death<br>मृत्यूचा दिनांक	□□/□□/□□□□<br>Days दिवस Month महिने Year वर्ष
Q0015	Place where death is registered<br>मृत्यूची नोंद कुठे केली?	01 Gram panchayat ग्रामपंचायत<br>02 Corporation महानगरपालिका<br>03 Others *(Specify)* इतर (स्पष्ट करा)<br>04 So far not registered अजून नोंद केलेली नाही
Q0016	Cause of death *(Record exactly what the respondent said)* मृत्यूचे कारण *(उत्तर देणाऱ्या व्यक्तीने जे/ जसे सांगितले असेल त्याची नोंद करा)*	__________________________________<br>__________________________________
Q0017	Is this death a maternal death? *(Ask this question only if the deceased is a woman who died during pregnancy or at the time of delivery or within 42 days of delivery)*<br>हा माता मृत्यू आहे का? *(मृत व्यक्ती ही गर्भधारणेदरम्यान किंवा बाळंतपणाच्या वेळी किंवा बाळंतपणाच्या 42 दिवसांच्या आत मरण पावलेली स्त्री असेल तरच हा प्रश्न विचारा)*	01 Yes होय<br>02 No नाही *(If no, stop questioning)* *(जर नाही असेल तर पुढील प्रश्न विचारू नका)*
Q0018	If yes, when did it happen? जर होय, तर कधी झाले?	01 During the pregnancy गर्भधारणेदरम्यान<br>02 At the time of delivery बाळंतपणाच्या दरम्यान<br>03 Within 42 days of delivery बाळंतपणाच्या 42 दिवसांच्या आत
Interview end time मुलाखत समाप्त होण्याची वेळ: □□:□□ *(24 hrs)*		

Death Event Form

Interviewer name: ________________ Signature: ___________ Date: □□/□□/□□□□

Data entry by __________________ Signature: _____________ Date: □□/□□/□□□□
